# Human α1-Antitrypsin Inhibits Nociceptor Excitability and Relieves Inflammatory and Neuropathic Pain

**DOI:** 10.3390/biom16081074

**Published:** 2026-07-23

**Authors:** Alaina L. Waters, Santiago Loya-López, Erick J. Rodriguez-Palma, Shainnel O. Eans, Ryosuke Shinouchi, Jordan Stokes, Jay P. McLaughlin, Sihong Song, Rajesh Khanna

**Affiliations:** 1 Department of Pharmacology and Therapeutics and Center for Advanced Pain Therapeutics and Research, College of Medicine, University of Florida, Gainesville, FL 32610, USA; 2 Department of Cellular and Systems Pharmacology, College of Pharmacy, University of Florida, Gainesville, FL 32610, USAjpmclaughlin@ufl.edu (J.P.M.); 3 Department of Pharmaceutics, College of Pharmacy, University of Florida, Gainesville, FL 32610, USAshsong@ufl.edu (S.S.)

**Keywords:** alpha-1 antitrypsin, nociception, dorsal root ganglion, T-type calcium channels, neuropathic pain, inflammatory pain

## Abstract

Chronic pain affects hundreds of millions of people and remains poorly managed, as the most effective drugs, including opioids, carry side effects that limit long-term use. Because inflammation drives both the initiation and maintenance of chronic pain, anti-inflammatory mechanisms are an attractive analgesic target. We investigated human alpha-1 antitrypsin (hAAT), a serine proteinase inhibitor with potent anti-inflammatory activity and established protection across disease models, as a candidate analgesic, using Aralast NP^®^, a clinical-grade, already-approved formulation that makes findings directly translatable. Using calcium imaging and patch-clamp electrophysiology in mouse dorsal root ganglion (DRG) neurons, we found that hAAT reduced activation of low-voltage-activated Ca^2+^ channels and dampened intrinsic excitability. Veratridine-evoked Ca^2+^ responses, a sodium-channel-dependent readout of nociceptor activity, were suppressed by hAAT to a degree comparable to the selective sodium channel inhibitors ProTx-II (Na_V_1.7) and VX-548 (Na_V_1.8), driven by loss of the nociceptor-associated response profiles. In vivo, hAAT decreased pain sensitivity and pain-associated behaviors in both inflammatory and neuropathic models. Together, these findings reveal a mechanism by which hAAT suppresses nociceptor activity and position Aralast NP^®^ as a safe, effective candidate for treating chronic pain.

## 1. Introduction

Chronic pain affects millions worldwide, including one in five U.S. adults [1]. It degrades quality of life, ranks among the leading causes of disability, and imposes a substantial socioeconomic burden [2]. It is frequently comorbid with mental health disorders such as anxiety and depression and with physical diseases including cardiovascular disease, diabetes, and arthritis, and it increases mortality when these conditions co-occur [3,4]. However, adequate treatments are lacking. Current options—nonsteroidal anti-inflammatory drugs (NSAIDs), opioids, and neuropathic pain medications—are often ineffective or carry severe side effects, including addiction, whose risk rises with the prolonged use that chronic pain demands [5].

Inflammation is highly correlated with chronic pain, and a growing body of evidence implicates inflammation as playing a key role in the development and maintenance of chronic pain [6,7,8]. Prolonged inflammation activates inflammatory pathways through the release of pro-inflammatory cytokines such as interleukins IL-6, IL-1β, and tumor necrosis factor alpha (TNF-α), as well as chemokines, prostaglandins, bradykinins, neuropeptides, and neurotrophic factors such as nerve growth factor (NGF) and brain-derived neurotrophic factor (BDNF). These mediators act on nociceptors, peripheral sensory neurons that transmit pain signals to the central nervous system, increasing their excitability and amplifying pain signaling [6,7,8]. This peripheral sensitization contributes not only to inflammatory pain, but also to neuropathic pain, where immune-cell activation and sustained neuroinflammatory signaling can maintain chronic pain.

Human alpha-1 antitrypsin (hAAT, also known as SERPINA1) is a 52 kD endogenous glycoprotein primarily synthesized in the liver. It is a member of the serine protease inhibitor (SERPIN) family and primarily functions to inhibit the activity of serine proteases such as neutrophil elastase, proteinase-3, and cathepsin G, which contribute to inflammatory responses by activation of pro-cytokines and by forming of damage-associated molecular patterns (DAMPs). hAAT also inhibits caspase 3 and prevents apoptosis. Beyond its anti-protease activity, hAAT suppresses the production of pro-inflammatory cytokines including IL-1β and TNF-α and increases anti-inflammatory cytokine IL-10 secretion [9,10]. hAAT has already been shown to be protective across multiple disease models, including diabetes, arthritis, lupus, and stroke, through multiple mechanisms, including the inhibition of tissue-damaging enzymes, the modulation of cytokine signaling, and the suppression of autoimmunity [11,12,13,14]. Human AAT—also known as alpha1-proteinase inhibitor and marketed as Aralast NP^®^—is already approved for clinical use [15]. The mechanistic studies described here were conducted using this same clinical-grade hAAT. As a result, these findings could translate rapidly to the clinical setting, bringing a potential new analgesic to the clinical doorstep with a substantially shortened development path.

Given the contribution of inflammation to chronic pain and the anti-inflammatory properties of hAAT, we posit that hAAT may present strong potential as a safe and effective therapeutic for the treatment of chronic pain. However, whether hAAT directly modulates primary sensory neuron excitability or translates into antinociceptive efficacy in persistent pain states remain unclear. Here, we examine how hAAT affects the excitability of nociceptive mouse DRG neurons and evaluate its antinociceptive potential across multiple mouse models of chronic pain.

## 2. Materials and Methods

### 2.1. Subjects

All experiments were performed using C57BL/6J mice from The Jackson Laboratory (Bar Harbor, ME, USA). Mice were housed three to five per cage and housed in ventilated acrylic cages under controlled environmental conditions including temperature (22 °C ± 1 °C), humidity (50%) and a 12/12 light–dark cycle. Food and tap water were provided ad libitum. All animal procedures were reviewed and approved by the University of Florida Institutional Animal Care and Use Committee (IACUC202400000002), and were conducted in accordance with the National Institutes of Health Guide for Care and Use of Laboratory Animals (Publication No. 85–23, revised 1985), as well as established ethical guidelines for experimental pain research in animals [16].

All animal studies are reported in compliance with the ARRIVE guidelines [17]. Final sample sizes (i.e., a fixed number of animals for a particular test) were predetermined by a power analysis statistical method, and animals were assigned to groups randomly. Drug treatment experiments were conducted in a blinded fashion. No animals were excluded from statistical analysis.

Several studies were conducted using single sexes of animals due to resource constraints; however, sexes were distributed across paradigms, ensuring that both sexes were evaluated.

### 2.2. Materials and Reagents

All chemicals, unless noted, were purchased from Sigma (St. Louis, MO, USA). Fura-2 AM (Cat#F1225) was obtained from Invitrogen (Waltham, MA, USA). TTA-P2 (Cat# HB5851) was obtained from Hello Bio (Bristol, UK) and maintained at −20 °C as 1 mM aliquots in DMSO to minimize freeze–thaw cycles. ProTx-II (Cat# 4023) was obtained from Tocris/Bio-Techne (Minneapolis, MN, USA), while VX-548 (Suzetrigine, Cat# HY-148800) was from MedChemExpress (Monmouth Junction, NJ, USA). Human alpha 1-antitrypsin (AAT), which is also known as alpha1-proteinase inhibitor and sold as Aralast NP^®^ [15], was obtained from Baxalta US Inc. (Lexington, MA, USA).

### 2.3. Preparation of Dissociated Dorsal Root Ganglion Neurons

DRGs were prepared as described by us previously [9,10]. Briefly, female C57BL/6J mice (~4–6 weeks) were anesthetized (5% isoflurane). All DRGs were collected, trimmed at their roots, and enzymatically digested in DMEM (Cat#11965; Thermo Fisher Scientific, Waltham, MA, USA) media with neutral protease (3.125 mg/mL; Cat# LS02106; Worthington, Lakewood, NJ, USA) and collagenase type I (5 mg/mL; Cat# LS004194; Worthington, Lakewood, NJ, USA) for 45 min at 37 °C under gentle agitation. The dissociated DRG neurons were gently centrifuged to collect cells and resuspended in complete DRG media (DMEM containing 1% penicillin/streptomycin sulfate from 10,000 µg/mL stock (Cat#15140; Life Technologies, Waltham, MA, USA) and 10% fetal bovine serum (Cat#FB12999105; Fisher Scientific, Waltham, MA, USA)). Cells were seeded on poly-D-lysine-coated coverslips.

### 2.4. Calcium Imaging

Changes in depolarization-induced calcium influx in mouse DRG neurons were assessed using a method previously published by us [18,19]. Briefly, neurons were loaded with 3 µM Fura-2AM for 30 min at 37 °C. A standard bath solution containing 139 mM NaCl, 3 mM KCl, 0.8 mM MgCl_2_, 1.8 mM CaCl_2_, 10 mM Na-HEPES, 5 mM glucose, pH 7.4, was used. Depolarization was evoked with 15 s pulses of 40 or 90 mM KCl with a 5 min washout period between triggers. hAAT (1 mg/mL) was added 3–5 min prior to baseline image acquisition and maintained throughout the washes and both trigger conditions, for a total exposure of approximately 15 min. The T-type channel selective blocker TTA-P2 (1 µM) [20] was used as a positive control in the stimulus with 40 mM KCl, and the non-selective Ca^2+^ channel blocker CdCl_2_ (20 µM) [21] was used as a positive control in the stimulus with 90 mM KCl. For the veratridine depolarization experiments, 30 μM veratridine was used as the stimulus in the same standard bath solution [22].

VTD-evoked responses were classified by their shape into three profiles, adapted from Mohammed et al. [23]. Oscillating (OS) responses were defined as two or more peaks (each ≥ 10% increase from baseline) separated by at least two frames (10 s) and with a return toward baseline (within 10% of baseline) between successive peaks; these criteria reduce false-positive peaks arising from noise and distinguish oscillating responses from slow-decay responses. Rapid-decay (RD) responses were defined as a single peak that returned to baseline within ≤120 s. Slow-decay (SD) responses were defined as a single peak that returned to baseline in >120 s, or that did not return to baseline by the end of the protocol; because recordings were not extended long enough to resolve intermediate-decay kinetics, intermediate-decay responses were grouped within the SD profile. Each of these categories is mutually exclusive, and when their percentages are combined with non-responders, they add up to 100% in every group.

Cells were first examined under bright-field to exclude those showing signs of lysis. Fluorescence imaging was achieved with an inverted microscope, Nikon Ts2R-FL (Nikon Instruments Inc., Melville, NY, USA), using a Nikon Fluor 4X and Hamamatsu ORCA-Fusion Gen-III sCMOS camera (Hamamatsu Photonics; Bridgewater, NJ, USA) controlled by NIS Elements AR software (version 6.20, Nikon Instruments). The excitation light was delivered by an AURA III light engine (Lumencor, Beaverton, OR, USA). The excitation filters (340 ± 5 nm and 380 ± 7 nm) were controlled by a Lumencor RETRA FURA filter cube. Fluorescence was recorded through a 505 nm dichroic mirror at 535 ± 25 nm. Images were taken every 5 s during the time course of the experiment to minimize photobleaching and phototoxicity and to provide acceptable image quality. Changes in [Ca^2+^]_c_ were monitored following a ratio of F340/F380, calculated after subtracting the background from both channels.

### 2.5. Measurement of Neuronal Excitability Using Whole-Cell Current-Clamp Electrophysiology

Electrophysiological recordings were conducted 15–24 h after plating using the whole-cell configuration of the patch-clamp technique in current-clamp mode [18]. Recordings were performed at RT (22–24 °C) using an EPC 10 amplifier (HEKA Elektronik; Germany) and acquired with PatchMaster (HEKA). For current-clamp recordings, the external solution contained (in millimolar): 154 NaCl, 5.6 KCl, 2 CaCl_2_, 1 MgCl_2_, 10 D-Glucose, and 8 HEPES (pH 7.4 adjusted with NaOH, and mOsm/L = 300). The internal solution was composed of (in millimolar): 137 KCl, 10 NaCl, 1 MgCl_2_, 1 EGTA, and 10 HEPES (pH 7.3 adjusted with KOH, and mOsm/L = 277). For the experimental condition, the external solution included 1 mg/mL hAAT. At RT (22–24 °C), a tight seal with the cell membrane was established by applying negative pressure to obtain a seal resistance >1 GΩ. A brief pulse of negative pressure was then applied to rupture the cell membrane and establish the whole-cell patch-clamp configuration. Neurons were initially held at −60 mV in voltage-clamp mode to measure seal quality before switching to current-clamp mode, where the current injection was immediately set to 0 pA to measure the resting membrane potential (RMP). DRG neurons with an RMP more hyperpolarized than −40 mV, stable baseline recordings, and evoked spikes that overshot 0 mV were used for experiments and analysis. Action potentials were evoked by current injection steps from 0 to 120 pA with an increment of 10 pA in 300 ms steps. Rheobase was measured by injecting currents from 0 pA with an increment of 10 pA in 50 ms steps.

Pipettes were pulled from standard wall borosilicate glass capillaries (Sutter Instrument; Novato, CA, USA) with a horizontal puller (Model P-97, Sutter Instrument). The resistance of the pipettes when filled with internal solution and immersed in the recording bath ranged from 2 to 4 MΩ. Recordings were performed from small DRG neurons with a maximum capacitance of 15 (<23 mm diameter). Series resistance under 7 MΩ was deemed acceptable. All experiments had a series resistance compensation between 60 and 90 were filtered at 10 kHz, and digitized at 10–20 kHz. Analyses were performed using Fitmaster software v1.7 (HEKA) and Origin 9.0 software (OriginLab).

### 2.6. Pain Models and Behavioral Testing

#### 2.6.1. LPS-Induced Hyperalgesia Assay (48 °C Warm Water Tail-Withdrawal Assay)

The nociceptive stimulus was 48 °C water, with latency to withdraw the tail taken as the endpoint. The water temperature of 48 °C was selected for this work to ensure a moderate tail-withdrawal response with a measurable decrease in withdrawal time possible, but also a significant temperature for hyperalgesic testing [24]. Animals showing an initial baseline latency of <4 s or >15 s were to be excluded from the study; notably, no subjects were excluded. A maximum response time of 30 s was used to prevent tissue damage. After determining control latencies, mice received a single i.p. dose of hAAT (30 mg/kg) and 5 min later, lipopolysaccharide (LPS; Sigma-Aldrich, St. Louis, MO, USA) (0.3 mg/kg, i.p.) was administered. LPS-induced hyperalgesia was induced using combined methods similar to previous reports [25,26], where decreases in tail-withdrawal latency indicate hyperalgesic effects. Data for hyperalgesic testing are reported in 10 min intervals after administration of LPS (or saline as a control) as average latency (in seconds, s) to withdraw the tail (s ± SEM) in 8–12 mice.

#### 2.6.2. Formalin Assay

Additional testing of antinociceptive potency against inflammatory pain was performed using the formalin (Sigma-Aldrich, St. Louis, MO, USA) assay in C57BL/6J mice as previously described [27]. Following a 5 min pretreatment of a single dose of vehicle control or test drug (i.p.), an intraplantar (i.pl.) injection of 5% formalin (2.5 μg in 15 μL) was administered into the right hind paw. Paw-licking duration was recorded in 5-minute intervals for 60 min following injection. The last 55 min was used to determine response to an inflammatory stimulus. Data were analyzed as area under the curve (AUC) representing summed time mice spent licking their inflamed hind paw. Saline treatment resulted in an AUC of 203.2 ± 32.1. Antinociception for each drug-treated mouse was calculated by comparing the test group to a control group in which mice were treated with the appropriate vehicle (saline, i.p.) using the formula:

% antinociception = ([{average AUC in the vehicle group} − {AUC of each test mouse}]/ [average AUC in vehicle group]) × 100.

#### 2.6.3. Neuropathic Pain Model

Neuropathic pain was induced using the spared nerve injury model, as previously described [28]. Briefly, mice were anesthetized with isoflurane (5% for induction, 2% for maintenance). Following surgical exposure of the sciatic nerve, the common peroneal and tibial branches were tightly ligated using 7-0 silk suture (BD, Canaan, CT, USA) and transected approximately 2 mm distal to the ligation site, while the sural nerve branch was left intact.

#### 2.6.4. Mechanical Allodynia

Mechanical sensitivity was assessed using the up–down method to determine the 50% paw withdrawal threshold. Mice were first habituated for 60 min in transparent acrylic cages placed on a mesh floor. After acclimation, calibrated von Frey filaments were applied to the plantar surface of the paw, and responses were recorded following the up–down method as previously described [29]. The 50% withdrawal threshold was calculated using the following formula:

50% Threshold (g) = (10^[Xf + κ*δ*]^/10,000)

where Xf represents the logarithmic value of the last von Frey filament applied, κ corresponds to a correction factor derived from the response pattern based on a calibration table, and *δ* indicates the mean difference between logarithmic stimuli intensities [29]. A withdrawal threshold below 0.2 g was considered indicative of mechanical allodynia.

#### 2.6.5. Cold Allodynia

Cold allodynia was evaluated using an acetone evaporation assay, as previously reported [30]. After completion of the von Frey test, 20 μL of acetone (Sigma-Aldrich, MO, USA) was gently applied to the lateral side of the injured hind paw using a syringe connected to PE-90 tubing (BD, Canaan, CT, USA). Behavioral responses—including licking, flinching, shaking, or lifting of the paw—were recorded over a 60 s period, and the total duration of these behaviors was defined as the aversive response to the cold stimulus.

### 2.7. Statistical Methods and Data Analysis

Graphing and statistical analysis were undertaken with GraphPad Prism (Version 11; GraphPad Software, Boston, MA, USA). Details of statistical tests, significance, and sample sizes are reported in the appropriate figure legends. All data plotted represent mean ± SEM with a significance set at *p* < 0.05 denoted by the asterisk (*). For electrophysiological recordings, data were compared using a Welch’s *t*-test (RMP), Mann–Whitney test (rheobase), or two-way ANOVA with Geisser–Greenhouse correction for sphericity, followed by Šídák-adjusted multiple comparisons (sensory neuron excitability). Linear regression was used to determine ED_50_ values, and 95% confidence intervals of dose–response curves were presented for the formalin assay. Significant differences in behavioral data were analyzed by ANOVA (one-way or two-way repeated measures), with significant results further analyzed with Tukey’s post hoc test as appropriate for significant pairwise comparisons between groups. AUC from time-course behavioral data with two groups was analyzed using Welch’s *t*-tests. All two-group comparisons are reported as two-tailed *p*-values.

## 3. Results

### 3.1. hAAT Inhibits LVA Ca^2+^ Ion Channels in Female Mouse DRGs

**Figure 1:**
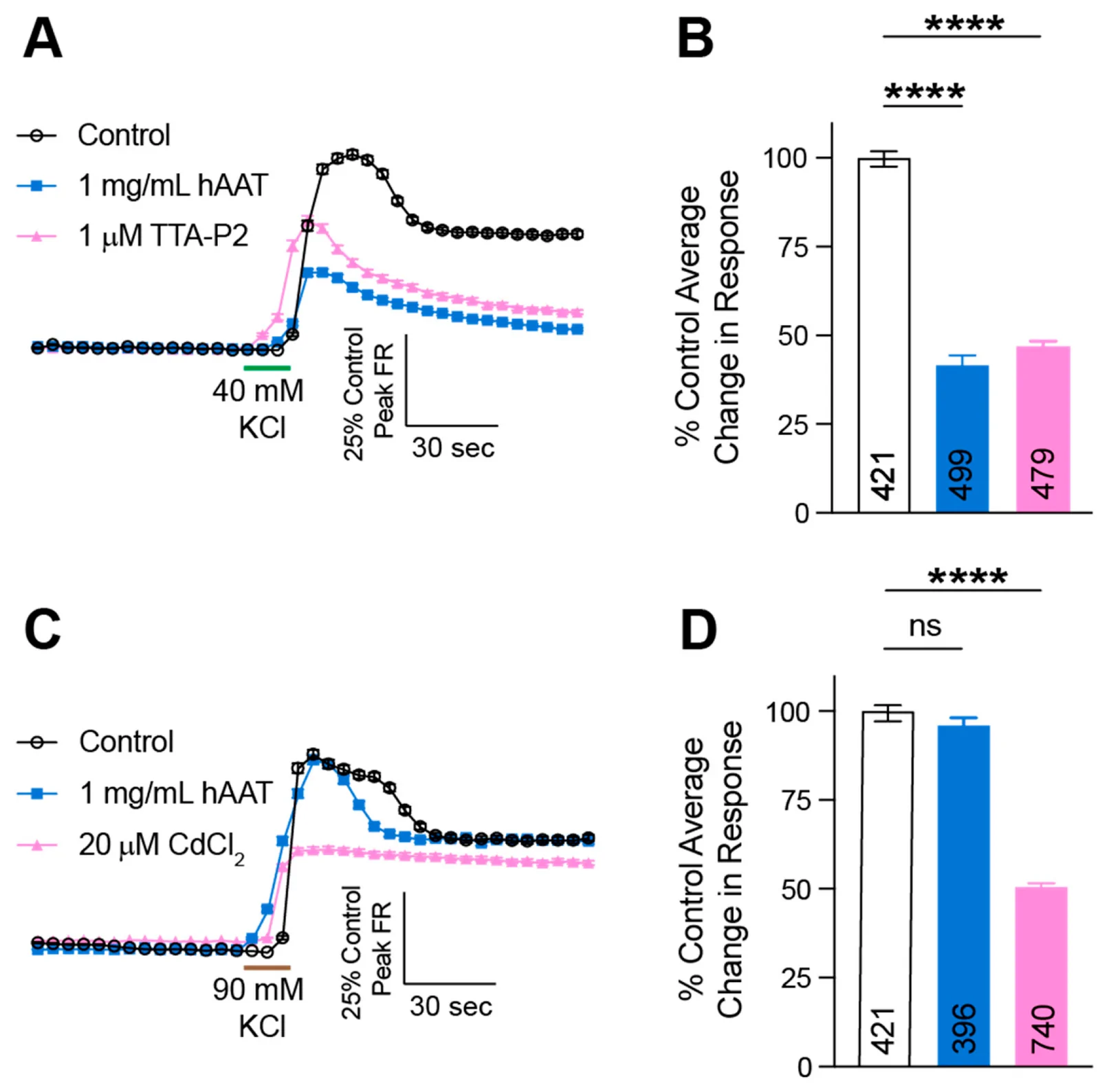
hAAT reduces intracellular calcium entry in response to 40 mM, but not 90 mM, KCl in DRG neurons from female mice. DRGs were collected from female mice, dissociated, and cultured overnight, then calcium entry was recorded via calcium imaging in response to 15 s 40 or 90 mM KCl. (**A**) Average percentage of peak same-day control response to a 15 s 40 mM KCl trigger calculated from cells in the presence of control solution (black, *n* = 421 from 3 independent coverslips), 1 mg/mL hAAT (blue, *n* = 499 from 4 independent coverslips), or 1µM TTA-P2 (pink, *n* = 479 from 3 independent coverslips). Error bars show SEM. (**B**) Average percentage of peak same-day control response to 15 s 40 mM KCl trigger. Error bars show SEM; **** *p* < 0.0001, one-way ANOVA, Tukey post hoc multiple comparisons. (**C**) Average percentage of peak same-day control response to 15 s 90 mM KCl trigger calculated from cells in the presence of control solution (black, *n* = 421 from 3 independent coverslips), 1 mg/mL (blue, *n* = 396 from 3 independent coverslips), or 20 µM CdCl_2_ (pink, *n* = 740 from 4 independent coverslips). Error bars show SEM. (**D**) Average percentage of peak same-day control response to 15 s 90 mM KCl trigger. Error bars show SEM; **** *p* < 0.0001, one-way ANOVA, Tukey post hoc multiple comparisons; ns, not significant.

Inflammatory processes are known to cause damage to DRG neurons and result in hyperexcitability [31,32]. To evaluate if hAAT influences the excitability of these neurons, we conducted calcium imaging of female mouse DRG neurons. Neurons pretreated with hAAT (1 mg/mL, 5 min) and then exposed to a 40 mM KCl trigger solution for 15 s exhibited decreased overall calcium entry and peak change in cytosolic calcium ([Ca^2+^]_c_) by 58.45% ± 1.77% (mean ± SEM) compared to control (Figure 1A,B), indicating a reduction in the response of low-voltage-activated (LVA) Ca^2+^ ion channels. The LVA blocker TTA-P2 inhibited ([Ca^2+^]_c_) by 51.24% ± 2.07% compared to control (Figure 1A,B).

Neurons exposed to hAAT and exposed to a 90 mM KCl trigger solution for 15 s showed no significant difference in overall calcium entry or peak change in [Ca^2+^]_c_ compared to control (Figure 1C,D), indicating that hAAT did not influence the response of high-voltage-activated (HVA) Ca^2+^ ion channels. The non-specific and HVA blocker CdCl_2_ inhibited ([Ca^2+^]_c_) by 50.12% ± 1.07% compared to control (Figure 1C,D). Together, these data indicate that hAAT has an inhibitory effect on the activity of female mouse DRG neurons, specifically by inhibiting the activation of LVA Ca^2+^ ion channels without affecting the activity of HVA Ca^2+^ ion channels.

**Figure 2:**
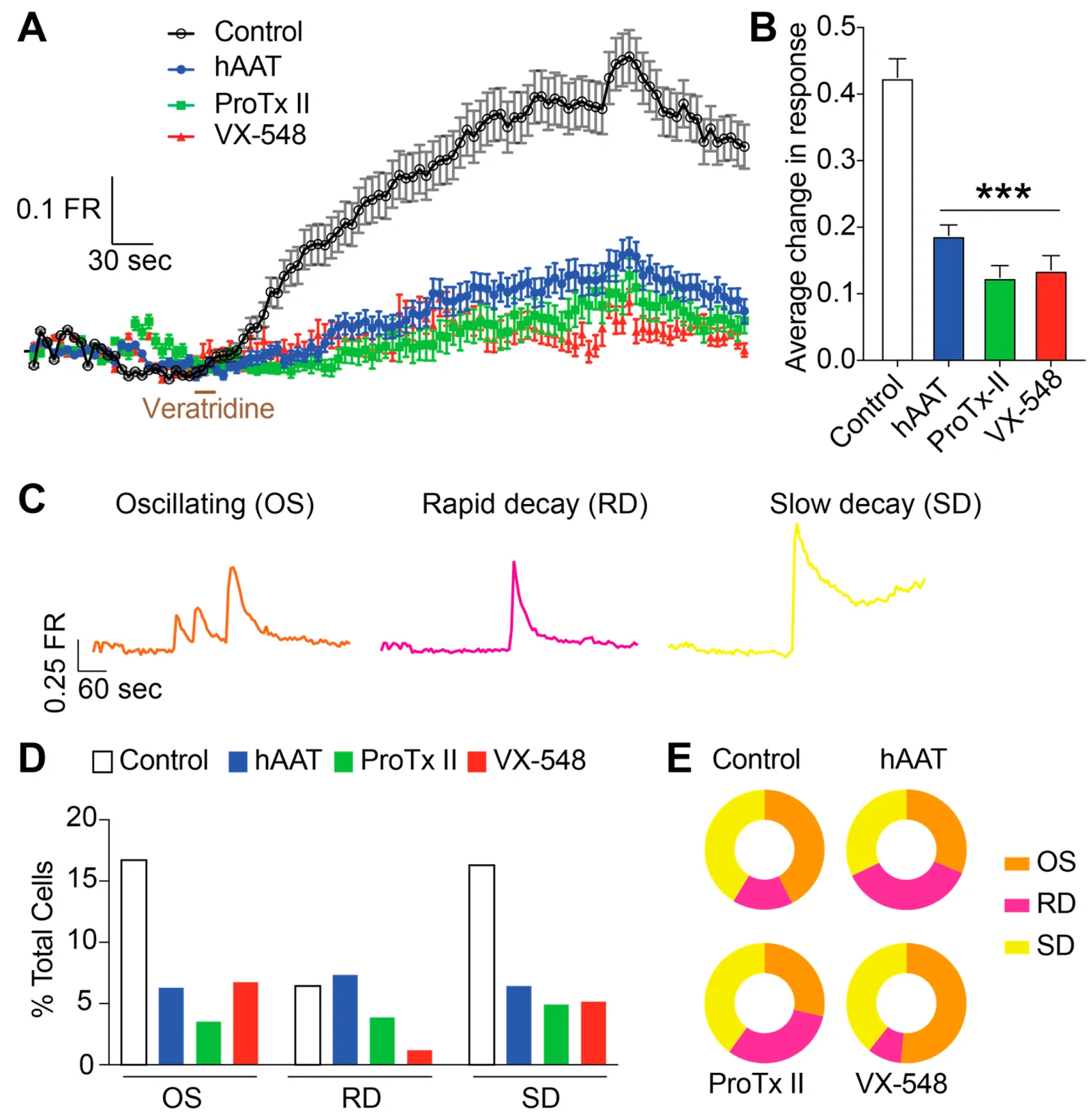
hAAT suppresses veratridine-evoked calcium responses in DRG neurons to a degree comparable to selective sodium channel blockers. (**A**) Representative averaged traces (mean ± SEM) of the change in intracellular calcium (Fura-2 fluorescence ratio, FR) evoked by veratridine (VTD) in cultured mouse DRG neurons under control conditions (black, open circles; *n* = 497 from 3 independent coverslips) and following pre-treatment with hAAT (blue; *n* = 667 from 3 independent coverslips), the Na_V_1.7-selective inhibitor ProTx-II (5 nM; green; *n* = 284 from 3 independent coverslips), or the Na_V_1.8-selective inhibitor VX-548 (5 µM; red; *n* = 252 from 3 independent coverslips). Bar denotes the VTD application period; scale bars 0.1 FR (vertical) and 30 s (horizontal). VTD is a voltage-gated sodium channel modifier [35] that results in depolarization, which drives secondary Ca^2+^ influx. (**B**) Mean change in VTD-evoked response amplitude for each condition. The VTD response was significantly reduced relative to control by all three treatments (*** *p* < 0.001 versus control by one-way ANOVA with Dunnett’s post hoc); the reduction produced by hAAT did not differ significantly from that produced by ProTx-II or VX-548. (**C**) Representative traces illustrating the VTD response profiles classified by response shape, after Mohammed et al. [23]: oscillating (OS, multi-peak), rapid decay (RD, transient peak resolving during application), and slow decay (SD, single peak with prolonged decay). Because recordings were not extended long enough to resolve intermediate-decay kinetics, intermediate-decay responses were grouped with the SD profile. (**D**) Percentage of total imaged cells displaying each response profile under each condition. (**E**) Proportional distribution of the three response profiles within the VTD-responsive population for each condition.

Veratridine (VTD) is a voltage-gated sodium channel modifier that binds open sodium channels and is widely used to activate DRG neurons in functional assays [23]. Because VTD-evoked calcium influx integrates the activity of the sodium, potassium, and calcium channels expressed in a given sensory neuron, it provides a sensitive functional readout of neuronal excitability. VTD evoked a slow, sustained rise in the F_340_/F_380_ ratio in control neurons that developed over several minutes, consistent with Na_V_-driven depolarization and downstream Ca^2+^ entry (Figure 2A, black). To establish the molecular basis of this signal, we applied subtype-selective Na_V_ blockers in the absence of hAAT: the Na_V_1.7-selective blocker ProTx II [33] and the Na_V_1.8-selective inhibitor VX-548 [30,34] each strongly attenuated the response (Figure 2A, green and red), confirming that the VTD-evoked Ca^2+^ signal is carried by Na_V_1.7 and Na_V_1.8 and validating the assay as a readout of these channels. Having established this dependence, we tested hAAT and found that it suppressed the response to a comparable degree (Figure 2A, blue). hAAT reduced the average change in response by ~57% relative to control (~0.42 → ~0.18; *** *p* < [0.001]; Figure 2B).

To determine whether hAAT acted broadly or on a defined neuronal subpopulation, we classified VTD responses by their shape into oscillating (OS), rapid-decay (RD), and slow-decay (SD) profiles (Figure 2C), following the scheme of Mohammed et al. [23]; intermediate-decay responses were grouped with SD because recordings were not extended long enough to resolve their slower kinetics. Across conditions, the reduction in responding cells was driven predominantly by loss of the OS and SD populations. The OS population fell from ~16.7% of total cells under control conditions to ~6.3% with hAAT, ~3.5% with ProTx-II, and ~6.7% with VX-548 and the SD population fell from ~16.3% in control conditions to ~6.4% with hAAT, ~3.5% with ProTx-II, and ~5% with VX-548 (Figure 2D). The RD population was reduced by ProTx-II and VX-548, but not by hAAT (control ~6.4%; hAAT ~7.3%; ProTx-II ~3.9%; VX-548 ~1.2%). Examining the proportional composition of the VTD-responsive population (Figure 2E) showed that this shift reflected a relative depletion of OS and SD responders following each treatment.

### 3.2. hAAT Decreases Evoked Action-Potential Firing Frequency of Female Mouse DRGs

**Figure 3:**
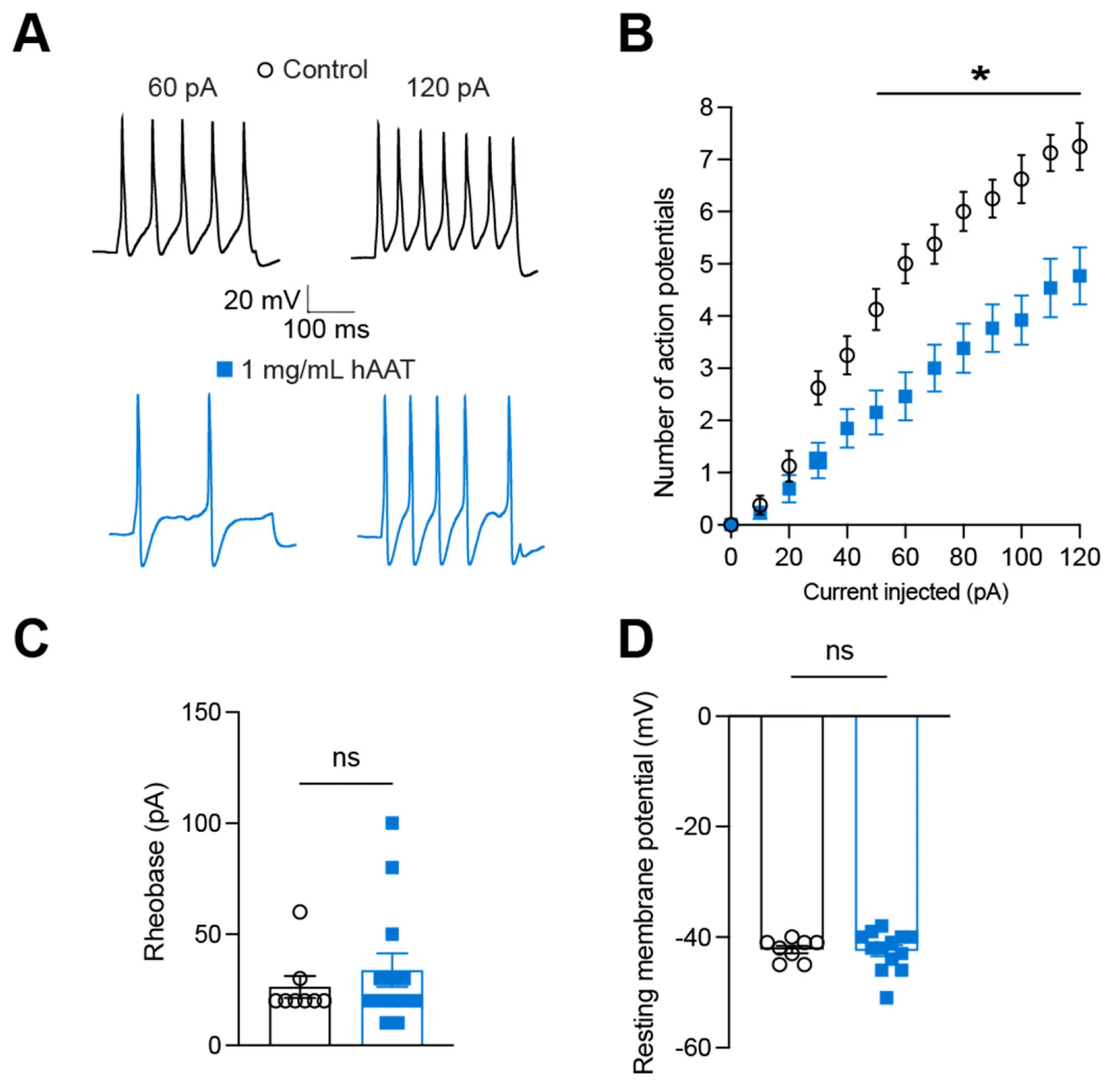
hAAT reduces action-potential firing frequency in response to applied current. DRGs were collected from female mice, dissociated, and cultured overnight, then rheobase, RMP, and evoked action-potential firing were measured using whole-cell patch-clamp electrophysiology. (**A**) Representative action-potential firing of DRGs in control solution (*black*) or 1 mg/mL hAAT (*blue*) evoked by 300 ms 60 and 120 picoamperes (pA) current stimulus. (**B**) Quantification of the number of evoked action potentials of DRGs in control solution (*n* = 8 cells) or 1 mg/mL (*n* = 13 cells) in response to 0–120 pA of injected current. Error bars show SEM; * *p* < 0.05, two-way ANOVA, Šídák post hoc multiple comparisons. (**C**) Average and individual values of rheobase of DRG neurons. Error bars show SEM; Mann–Whitney test. (**D**) Average and individual values of RMP of DRG neurons. Error bars show SEM; Welch’s *t*-test. ns, not significant.

To determine whether hAAT modulates the excitability of mouse nociceptive DRG neurons, we performed whole-cell patch-clamp recordings on cultured small-diameter DRG neurons isolated from female mice. Resting membrane potential (RMP), rheobase, and action-potential (AP) firing frequency in response to stepwise depolarizing currents were assessed. Exposure to hAAT (1 mg mL^−1^, 3–5 min) produced a significant reduction in AP firing frequency beginning at an injected current of 50 pA compared with vehicle-treated controls (*p* < 0.05; Figure 3A,B). By contrast, neither rheobase nor RMP differed between hAAT-treated and control cells (*p* > 0.05; Figure 3C,D). These findings indicate that hAAT selectively attenuates stimulus-evoked firing of nociceptive DRG neurons without altering their basal electrophysiological properties or the threshold current required for AP initiation.

### 3.3. hAAT Reduces LPS- and Formalin-Induced Inflammatory Pain

**Figure 4:**
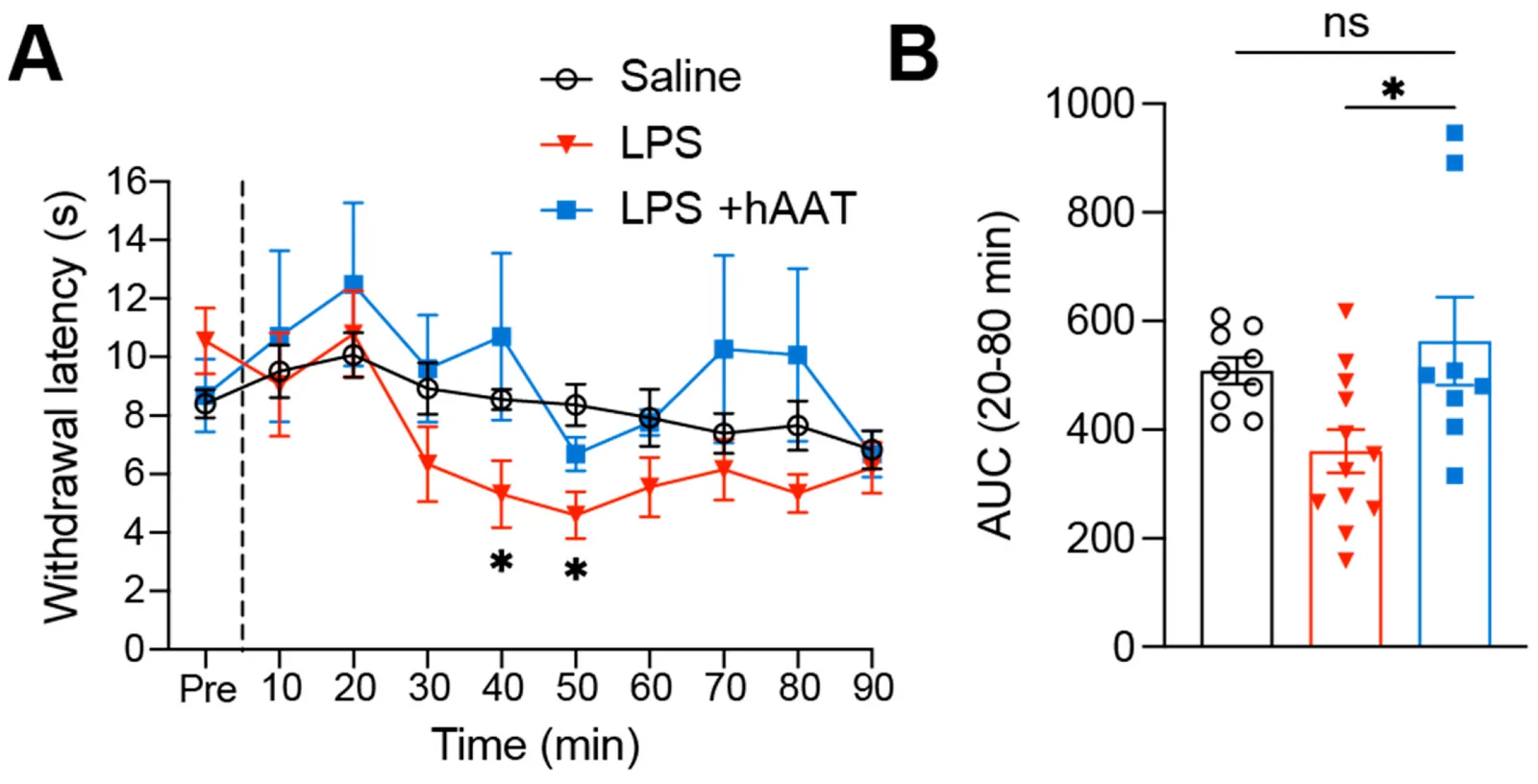
hAAT pretreatment prevents LPS-induced hyperalgesia in the mouse 48 °C warm water tail-withdrawal assay. Control mice were treated with saline (0.9%; black, *n* = 9 mice) or LPS (0.3 mg/kg; red, *n* = 12 mice). Test mice (blue, *n* = 8) were pretreated with hAAT (30 mg/kg) 5 min before LPS. (**A**) Time course showing effects of LPS ± hAAT on tail-withdrawal latency from 48 °C water before (pre) and every ten minutes after treatment. Error bars show SEM; * *p* ≤ 0.05 LPS vs. saline, two-way ANOVA, Tukey post hoc multiple comparisons. (**B**) Area under the curve (AUC) analysis from 20 to 80 min for withdrawal latency. Error bars show SEM; ns, no significant difference; * *p* < 0.05, one-way ANOVA, Tukey post hoc multiple comparisons.

Next, we assessed whether hAAT influences pain sensitivity in vivo in mouse models of inflammatory pain. First, mice were injected intraperitoneally (IP) with saline or hAAT (30 mg/kg). After five minutes, to trigger inflammatory pain, hAAT-injected mice and a subset of saline-injected mice were injected with lipopolysaccharide (LPS; 0.3 mg/kg), a component of bacterial cell walls known to cause inflammation and immune activation [36]. Tail-flick latency was then measured at 10 min intervals using a 48 °C water bath. Whereas mice treated with saline alone showed no significant difference from baseline withdrawal latencies over 90 min (Figure 4), LPS treatment (0.3 mg/kg) resulted in a significant reduction in tail-withdrawal latency compared to the saline response over time, particularly at 40 and 50 min (Figure 4A). In contrast, mice pretreated 5 min prior to LPS with hAAT (30 mg/kg) demonstrated no significant differences from the saline response across time (Figure 4).

**Figure 5:**
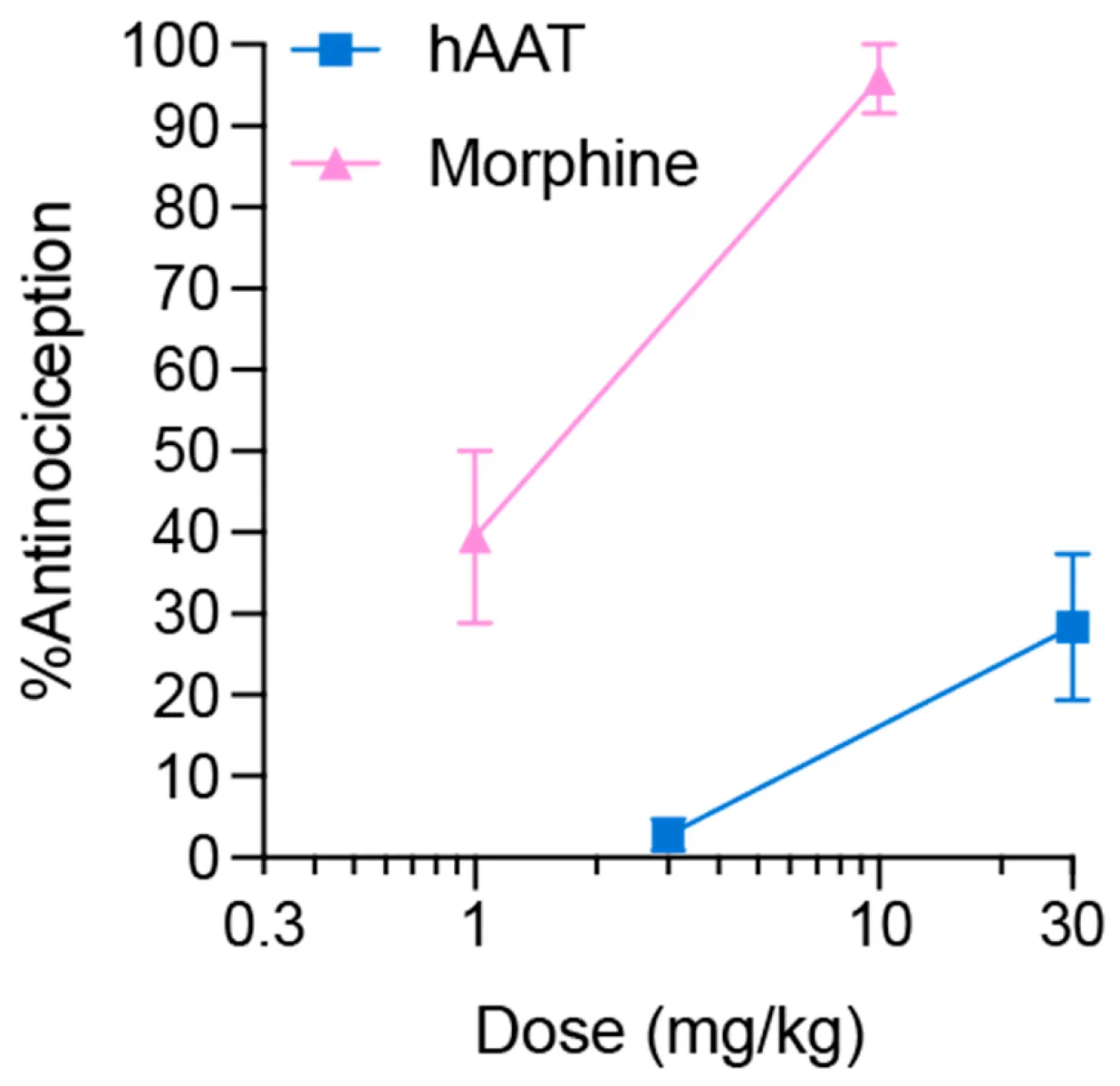
Dose-dependent antinociception of morphine and hAAT in Phase II of the mouse formalin assay. C57BL/6J mice received a single intraperitoneal (i.p.) dose of hAAT (0.3 or 30 mg/kg; blue), morphine (1 or 10 mg/kg; pink), or vehicle control (0.9% saline, i.p.) 5 min before intraplantar injection of 5% formalin into the right hind paw. Paw-licking duration was recorded over 60 min, and the last 55 min was summed as area under the curve (AUC) to quantify the response to the inflammatory stimulus. The y-axis, % antinociception, expresses each test mouse’s response relative to the saline control group, calculated as % antinociception = ([mean AUC of the vehicle group − AUC of the test mouse]/mean AUC of the vehicle group) × 100. Higher values therefore indicate greater pain relief (less paw-licking), and the vehicle group averages 0% by definition. Error bars show SEM. *n* = 10 animals per group.

We also examined the effect of hAAT in a second mouse model of inflammatory pain: a formalin (5%, 10 µL) intraplantar injection in a hind paw to induce a biphasic pain response. Phase I is thought to reflect direct activation of peripheral sensory neurons, whereas the prolonged Phase II reflects ongoing nociceptive input, inflammatory mediator signaling, and activity-dependent central sensitization in the spinal cord [37]. Accordingly, we assessed time spent licking the affected paw during Phase II to evaluate antinociceptive response against inflammatory pain. Consistent with previous reports, morphine treatment proved efficacious, with an ED_50_ (and 95% C.I.) value of 1.54 (0.68–2.54) mg/kg [38]. By comparison, hAAT treatment resulted in analgesia in the Phase II formalin response with 30 mg/kg hAAT having equivalent efficacy to the 1 mg/kg dose of morphine (Figure 5).

### 3.4. Reversal of Allodynia by hAAT in the Spared Nerve Injury (SNI) Model of Neuropathic Pain

To determine whether hAAT affects pain-related behavior in a model of chronic pain, we evaluated hAAT in male mice with neuropathic pain induced by the spared nerve injury (SNI) model. Mice received hAAT (10 mg/kg), and mechanical and cold sensitivity were assessed over a 24 h time course.

**Figure 6:**
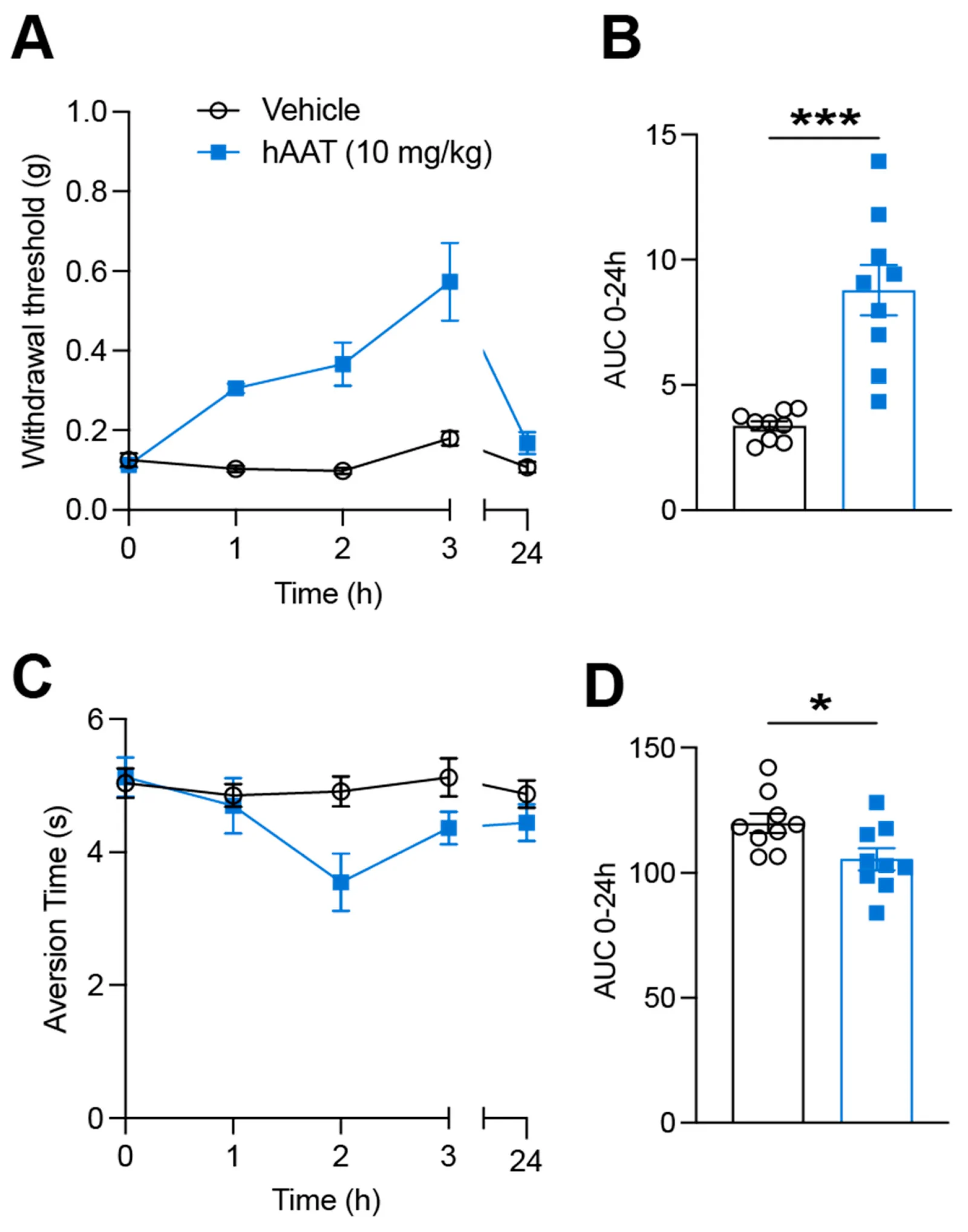
Intraperitoneal hAAT treatment reduces mechanical and cold hypersensitivity in male mice with neuropathic pain. Male mice with neuropathic pain received intraperitoneal administration of hAAT at 10 mg/kg or vehicle, and pain-like behaviors were assessed over a 24 h time course. (**A**) Time course showing the effect of hAAT on mechanical allodynia, measured as paw withdrawal threshold using von Frey filaments. (**B**) Area under the curve (AUC) analysis from 0 to 24 h for mechanical withdrawal thresholds. *** *p* < 0.001, Welch’s *t*-test. (**C**) Time course showing the effect of hAAT on cold allodynia, measured as aversion time in the acetone test. (**D**) AUC analysis from 0 to 24 h for cold aversion time. * *p* < 0.05, Welch’s *t*-test. Error bars show SEM. Nine animals per group.

Intraperitoneal administration of hAAT increased mechanical withdrawal thresholds compared with vehicle-treated mice, indicating a reduction in mechanical allodynia. This effect was evident within the first hour after treatment, increased over the three-hour post-administration period, and returned toward baseline levels by 24 h after administration (Figure 6A). Analysis of the area under the curve from 0 to 24 h confirmed a significant increase in mechanical withdrawal threshold following hAAT treatment compared with vehicle (Figure 6B). hAAT also reduced cold allodynia, measured as aversion time in the acetone test (Figure 6C,D). Although this effect was more modest than that observed for mechanical sensitivity, hAAT-treated mice showed lower aversion time during the early post-treatment period compared with vehicle-treated mice (Figure 6C). Consistent with this observation, AUC analysis from 0 to 24 h showed a significant reduction in cold aversion time after hAAT administration (Figure 6D).

Together, these data suggest that hAAT produces antinociceptive effects in male mice, with a stronger effect on mechanical allodynia and a more modest but significant effect on cold hypersensitivity.

## 4. Discussion

The principal finding of this study is that human α1-antitrypsin (hAAT) reduces primary sensory neuron excitability and produces antinociceptive effects across inflammatory and neuropathic pain states. Three lines of evidence converge on this conclusion. First, hAAT selectively suppressed low-voltage-activated (LVA) Ca^2+^ entry in dissociated female mouse dorsal root ganglion (DRG) neurons while leaving high-voltage-activated (HVA) Ca^2+^ entry intact. Second, hAAT reduced the gain of evoked action-potential (AP) firing in small-diameter nociceptors without altering passive membrane properties or spike threshold. Third, systemically administered hAAT prevented LPS-induced thermal hyperalgesia, produced antinociception in the formalin model with efficacy comparable to 1 mg/kg morphine, and reversed both mechanical and cold hypersensitivity in the spared nerve injury (SNI) model of neuropathic pain. Taken together, these data extend the biology of hAAT beyond its canonical role as a circulating anti-protease and identify it as a candidate endogenous modulator of nociceptor excitability and a translatable analgesic. Importantly, this mechanism is identified empirically at the cellular level rather than extrapolated from hAAT’s canonical anti-protease activity, though the upstream molecular event—and whether it depends on protease inhibition—remains to be defined. Critically, this represents a mechanism that is directly identified at the cellular level rather than inferred from hAAT’s canonical anti-protease and anti-inflammatory functions, providing a defined nociceptor target that links our in vitro and in vivo observations.

### 4.1. A Physiological Rationale for a Neuronal Role of hAAT

hAAT is the most abundant serine protease inhibitor in human plasma and the prototypic member of the serpin superfamily [39,40]. It is classically defined by inhibition of neutrophil elastase via its reactive center loop, and intravenous plasma-derived hAAT is FDA-approved as augmentation therapy in α1-antitrypsin deficiency. However, the now-substantial literature establishes that hAAT possesses pleiotropic anti-inflammatory and immunomodulatory activities that are at least partially independent of protease inhibition [41,42,43,44]. hAAT binds IL-8 and interferes with CXCR1-dependent neutrophil chemotaxis, lowers the production of the prototypical pronociceptive cytokines TNF-α, IL-1β, and IL-6, suppresses NF-κB activation, and engages an array of non-protease partners including low-density lipoprotein receptor-related protein 1 (LRP1), gp96, heat shock protein 70 (HSP70), and lipid-raft components [43,44,45]. Of relevance to the present work, hAAT has been reported to attenuate Toll-like receptor 4 (TLR4) signaling: it reduces LPS-driven activation of macrophages and dendritic cells and downregulates surface TLR4 and TLR2 expression [14,41,46,47]. hAAT is also not solely a hepatic product; it is synthesized locally by monocytes, macrophages, microglia, neutrophils, and epithelial cells, positioning the protein to act within tissue microenvironments that include peripheral nerve and ganglia [48,49]. These features make a direct or microenvironmental role for hAAT in sensory neurobiology biologically plausible rather than incidental, and they frame our electrophysiological and behavioral findings as the functional readout of an endogenous protein with under-recognized neuromodulatory capacity.

### 4.2. Selective Inhibition of LVA (T-Type) Calcium Entry as a Candidate Mechanism

The calcium-imaging data provide the clearest mechanistic signature of hAAT action. A mild depolarizing stimulus (40 mM KCl), which preferentially recruits LVA T-type channels, produced a calcium response that was reduced by hAAT. In contrast, hAAT did not affect calcium entry evoked by stronger depolarization with 90 mM KCl, a condition expected to recruit HVA calcium channels. The pharmacological controls reinforce this interpretation: the effect of hAAT phenocopied the selective T-type blocker TTA-P2 under mild depolarization, while the HVA blocker CdCl_2_ defined the channel population that hAAT did not engage. This pattern points to functional inhibition of LVA, most plausibly Cav3.2 channels.

Cav3.2 is the dominant T-type isoform in nociceptive DRG neurons and is a well-validated pain target. Genetic silencing or knockout of Cav3.2 produces antinociceptive, anti-hyperalgesic, and anti-allodynic phenotypes; Cav3.2-null mice show attenuated formalin responses; and selective T-type antagonists such as TTA-P2 are antinociceptive in inflammatory and neuropathic pain [20,50,51,52,53]. Because T-type channels activate near resting potential, generate low-threshold calcium spikes, and amplify burst firing, they set the excitability gain of nociceptors and are upregulated in injury and inflammation [53,54]. hAAT-mediated suppression of this conductance therefore offers a parsimonious mechanism that links our in vitro and in vivo observations.

An important nuance is that hAAT reduced evoked AP firing frequency from 50 pA upward without changing rheobase or resting membrane potential. If hAAT acted purely by removing a threshold-lowering T-current, a rightward shift in rheobase might have been expected. The dissociation we observe instead suggests that the dominant functional consequence is a reduction in suprathreshold firing gain rather than a change in the single-spike threshold—consistent with a contribution of T-type (and possibly other) conductances to repetitive firing and signal amplification, rather than to spike initiation alone. This interpretation reconciles a selective reduction in LVA-evoked calcium entry with preserved passive properties, but it also constrains the strength of the mechanistic claim: our data demonstrate functional LVA inhibition without establishing direct channel modulation. Isolated T-current recordings, Cav3.2-selective tools or knockout neurons, and binding/competition studies will be required to determine whether hAAT modulates Cav3.2 directly, acts through a receptor-coupled signaling cascade (for example via LRP1, which is abundantly expressed in neurons and Schwann cells), or both.

Because excitability was reduced as a decrease in evoked firing frequency rather than a change in rheobase or resting membrane potential (RMP), the voltage-gated sodium (Na_V_) channels that set spike threshold and resting excitability—principally Na_V_1.7 (SCN9A) and the persistent, near-threshold current carried by Na_V_1.9 (SCN11A)—are unlikely to be the primary effectors, since their modulation would be expected to shift rheobase and/or RMP [55]. The firing-gain phenotype is more consistent with reduced availability or function of channels that carry the inward current during sustained, high-frequency discharge, most notably the TTX-resistant channel Na_V_1.8 (SCN10A), which dominates the action-potential upstroke in nociceptors, supports repetitive firing during maintained depolarization, and is a key contributor to cold-evoked excitability. Our calcium-imaging data are consistent with this interpretation: veratridine-evoked Ca^2+^ entry was strongly attenuated by the Na_V_1.8-selective inhibitor VX-548 and by the Na_V_1.7-selective blocker ProTx II, confirming the response as a Na_V_1.7/Na_V_1.8-dependent readout, and hAAT suppressed this response by ~57%. This suppression was driven predominantly by loss of the oscillating (OS) and slow-decay (SD) response profiles, while the rapid-decay (RD) profile was largely unaffected. The OS and RD profiles are the veratridine response shapes enriched in nociceptors, while the SD profile is enriched in non-nociceptors [23]. The inhibitory effects of hAAT on the OS and SD profiles were similar to those induced by ProTx II and VX-548, further supporting hAAT as an inhibitor of Na_V_1.7/Na_V_1.8 channels. Engagement of a Na_V_1.8-dependent pathway aligns with the firing-gain phenotype, whereas the concurrent reduction in Na_V_1.7-dependent influx—in the absence of a shift in rheobase or RMP—suggests that any action on Na_V_1.7 is not the dominant contributor to the change in excitability. A contribution from Na_V_1.6, which supports high-frequency firing at nodes and somata [56], cannot be excluded. Importantly, T-type (Cav3.2) and Na_V_ channels are functionally coupled—LVA calcium influx amplifies subthreshold depolarization and can enhance Na_V_ recruitment—so the LVA suppression we observe may indirectly curtail sodium-dependent repetitive firing rather than requiring a direct action on Na_V_ channels themselves. This same coupling tempers interpretation of the veratridine result, since the downstream Ca^2+^ rise depends on voltage-gated calcium channels including Cav3.2; the hAAT-mediated suppression we observe is therefore consistent with either a Na_V_-proximal action or the LVA suppression documented above. Direct voltage-clamp measurement of TTX-resistant sodium currents in future studies will be needed to resolve the relative contributions of each subtype and to distinguish a direct action on Na_V_ channels from an indirect effect mediated by Cav3.2.

### 4.3. Convergent Antinociception in Inflammatory Pain

The in vivo inflammatory pain results are consistent with, and mechanistically complementary to, the neuronal data. hAAT pretreatment fully prevented LPS-induced reductions in tail-withdrawal latency. LPS sensitizes nociceptors through TLR4, expressed on immune cells, glia, and sensory neurons, and drives release of TNF-α, IL-1β, and IL-6—mediators that both sensitize nociceptors acutely and upregulate Cav3.2 in DRG neurons [54,57]. Because hAAT both restrains TLR4 signaling/cytokine output [14,41,46] and directly suppresses LVA calcium entry, it is positioned to act at two convergent nodes of the same pathway: damping the inflammatory drive and the neuronal substrate that drive transduces.

The formalin data extend this logic. hAAT relieved the tonic, inflammatory phase of the formalin response but not the acute phase I. Because hAAT only partially suppresses LVA calcium and Na_V_ currents, it is better positioned to dampen the sensitization and repetitive firing that drive phase II than to abolish the intense, direct nociceptor activation of phase I, indicating that hAAT acts preferentially on inflammatory and central sensitization-related components of pain rather than on acute nociceptive signaling.

This mirrors prior work showing that the AAT carboxy-terminal peptide SP16, an LRP1 agonist, dose-dependently attenuates both phases of the formalin response and prevents nerve injury allodynia [51,58]. The agreement between full-length hAAT here and an AAT-derived LRP1 ligand strengthens the case that the analgesic activity is an intrinsic property of the molecule and its signaling partners rather than an artifact of a particular preparation. These results also align with Kaneva et al., who reported that both intra-articular and systemic AAT reversed joint inflammation and the associated nociception, alongside cartilage degradation, in the K/BxN serum-transfer and neutrophil-elastase models of arthritis [59]. Notably, that study identified AAT endogenously during the resolution phase of acute inflammation and linked it to pro-resolution, chondroprotective signaling—promoting chondrogenic gene expression (*col2a1*, *acan*, *sox9*) via PKA/CREB while suppressing Wnt/β-catenin and the matrix-degrading metalloproteinase enzymes mmp13 and adamts5—reinforcing the view that AAT acts as an endogenous, pain-relevant resolution mediator rather than a passive anti-protease [59].

We chose the LPS and formalin paradigms to probe inflammatory pain from complementary angles. Systemic LPS drives hyperalgesia through broad innate immune activation [60], while intraplantar formalin delivers a localized inflammatory stimulus whose prolonged nocifensive response reflects ongoing peripheral input, inflammatory signaling, and activity-dependent spinal sensitization [37]. Together, they let us test whether hAAT is antinociceptive across distinct inflammatory contexts and examine its direct effect on sensory neuron excitability. Carrageenan and complete Freund’s adjuvant, by contrast, produce more sustained localized inflammation with prolonged mechanical and thermal hypersensitivity [61], addressing a related but separate question: whether hAAT also suppresses the maintenance of persistent peripheral inflammatory pain. Testing these models is an important next step.

### 4.4. Reversal of Neuropathic Hypersensitivity

In the SNI model, systemic hAAT reversed mechanical allodynia strongly and cold allodynia more modestly, with a time course that peaked within a few hours and returned toward baseline by 24 h. This profile is consistent with a reversible, pharmacodynamically limited action of a circulating protein rather than disease modification, which is attractive for on-demand symptom control but implies that sustained benefit would require repeated or continuous dosing. The mechanistic fit is again strong: Cav3.2 accumulates in the uninjured sural nerve after SNI and contributes to mechanical allodynia, a process driven in part by inflammatory mediators (IL-6, IL-1β) released from activated macrophages and satellite glia [54]. An agent that both lowers these mediators and suppresses LVA calcium entry would be expected to act preferentially on the mechanical component, as observed. The smaller effect on cold allodynia is notable given that AAT (Prolastin) ameliorates cold hyperalgesia in sickle cell disease models [62], and suggests that the cold modality may depend more heavily on parallel pathways (e.g., TRP channel-mediated transduction) that hAAT engages less efficiently. The broader translational context is encouraging: AAT-derived strategies reduce neuroinflammation and pain behaviors in chemotherapy-induced peripheral neuropathy and improve outcomes in models of peripheral neuropathy such as Charcot-Marie-Tooth type 1A, where hAAT lowered circulating IL-6 and inhibited ADAM-17 [63].

### 4.5. Sex as a Biological Variable

A deliberate strength and an interpretive caveat of this study is its coverage of both sexes across paradigms: the calcium imaging and electrophysiology experiments used female mice, whereas the LPS, formalin, and SNI experiments used male mice. We distributed the sexes across paradigms so that both sexes are represented in the overall body of evidence. Pain mechanisms are sexually dimorphic, particularly in the immune contribution to chronic pain—spinal microglia, P2X4/BDNF, and TLR4 signaling predominate in males, whereas adaptive (T-cell) mechanisms predominate in females [64]. That hAAT was efficacious in female inflammatory paradigms and in a male neuropathic paradigm argues that its antinociceptive action is unlikely to be confined to a single sex-restricted immune pathway and is compatible with a sex-independent neuronal mechanism such as direct suppression of nociceptor LVA calcium entry. Nonetheless, because no single paradigm was tested in both sexes here, this conclusion is cautionary. Determining whether hAAT’s neuronal and behavioral effects are quantitatively equivalent in males and females is an important and tractable next step.

### 4.6. Translational Implications and Physiological Interpretation

hAAT is an endogenous acute-phase protein with a long clinical safety record and established human dosing, which lowers the barrier to repurposing it, or AAT-derived ligands, for pain and suggests that the risk of adverse effects in the treatment of chronic pain would be low. The combination of a direct neuronal action (LVA calcium suppression) with a well-characterized anti-inflammatory action is well matched to inflammatory and mixed pain states, where peripheral sensitization and neuroimmune signaling co-drive the phenotype. Serpins are primarily known for their anti-protease activity [65], and because proteolysis differentially regulates ion channel activity [66], hAAT may act as a functional inhibitor of ion channel activity in the peripheral nervous system.

More broadly, our findings invite a reframing of hAAT’s physiology: beyond protecting tissue from unopposed proteolysis, endogenous AAT may serve as a homeostatic brake on nociceptor excitability and neuroinflammation. AAT deficiency itself is not reported to induce hyperalgesia in patients; however, the observation that serpins are downregulated in the DRG in disease states associated with severe pain [67], together with local AAT production by macrophages and microglia [48,49], is consistent with an endogenous, microenvironmentally regulated analgesic tone that exogenous hAAT augments at pharmacologic or supra-physiologic levels.

In this study, we tested the inhibitory effects of hAAT in C57BL/6 mice, which express five mouse AAT isoforms. The genes encoding these isoforms (*Serpina1a–e*, each ~11 kb) are clustered on chromosome 12 and share highly conserved sequences, including introns [68]. Their divergent reactive center loop sequences direct each isoform toward different proteinases [69], though their precise functions remain unclear. Although our experiments demonstrate an effect of hAAT, the endogenous mouse isoforms may also contribute. Future studies should therefore use a mouse AAT-knockout model, in which all mouse AAT genes are deleted by CRISPR/Cas9, to exclude this possibility [70].

### 4.7. Limitations and Future Directions

Several limitations temper these conclusions. The molecular target of the LVA effect is inferred pharmacologically rather than demonstrated directly; isolated T-current recordings, Cav3.2-selective genetic tools, and binding or competition studies are needed to confirm the speculated Cav3.2 involvement and to distinguish direct channel modulation from receptor-mediated signaling. Whether the antinociceptive activity requires hAAT’s anti-protease function, or instead depends on non-protease domains and partners (notably LRP1), remains to be resolved, as does the relative contribution of peripheral versus central sites of action. Direct assessment of the neuroimmune response (macrophage and glial activation) [67,71] and of local elastase activity with and without hAAT will be particularly informative, as a dissociation between behavioral efficacy and protease inhibition would distinguish hAAT’s direct neuronal action from its canonical anti-elastase function. The reversible, short-lived behavioral effects highlight the need for pharmacokinetic characterization and for testing alternative formulations (recombinant, mutant, or peptide-based) that may retain or enhance neuromodulatory activity. Encouragingly, engineered long-acting variants are already advancing clinically: the recombinant AAT-Fc fusion protein INBRX-101 was well tolerated in a phase 1 trial and sustained functional AAT above target with extended every-3-to-4-week dosing, offering a plausible route to overcome the short duration of action observed here [72]. The anti-nociceptive effects we observed occur at pharmacologic (supra-physiologic) levels of AAT rather than at physiologic levels, consistent with the fact that AAT-deficient patients do not exhibit hyperalgesia and that the models used here are not mAAT-deficient. Finally, both sexes should be evaluated across all paradigms, and additional modalities and chronic dosing regimens examined. Because our study aimed to establish hAAT’s antinociceptive activity across mechanistically distinct pain states rather than to exhaustively survey inflammatory pain models, we did not examine the sustained localized inflammation produced by carrageenan or CFA—an important direction for future study. Addressing these questions will clarify whether hAAT represents a genuinely novel, mechanism-defined analgesic and will sharpen our understanding of the physiological roles this abundant serpin plays within the nervous system.

## 5. Conclusions

This study identifies human α1-antitrypsin as a modulator of sensory neuron excitability and a broadly effective antinociceptive agent across tested inflammatory and neuropathic pain models. hAAT suppressed low-voltage-activated calcium entry and reduced evoked firing in nociceptors without altering passive membrane properties, and it attenuated pain behaviors across inflammatory (LPS, formalin) and neuropathic (spared nerve injury) models in both sexes. These findings extend hAAT biology beyond protease inhibition, positioning this abundant, clinically approved serpin as a mechanism-defined candidate for repurposing in pain. Defining its molecular target and site of action is the essential next step.

## Data Availability

The original contributions presented in this study are included in the article. Further inquiries can be directed to the corresponding author.

## Abbreviations

The following abbreviations are used in this manuscript:

AAT	α_1_-antitrypsin
ADAM-17	a disintegrin and metalloproteinase 17
ANOVA	analysis of variance
AP	action potential
ARRIVE	Animal Research: Reporting of In Vivo Experiments (reporting guidelines)
AUC	area under the curve
BDNF	brain-derived neurotrophic factor
[Ca^2+^]c	cytosolic (intracellular) calcium concentration
Cav3.2	low-voltage-activated T-type calcium channel, Cav3.2 isoform
CdCl_2_	cadmium chloride (non-selective HVA calcium channel blocker)
C.I.	confidence interval
CREB	cAMP response element-binding protein
CXCR1	CXC chemokine receptor 1
DAMPs	damage-associated molecular patterns
DMEM	Dulbecco’s Modified Eagle Medium
DMSO	dimethyl sulfoxide
DRG	dorsal root ganglion (ganglia)
ED_50_	median effective dose
FDA	(U.S.) Food and Drug Administration
gp96	glycoprotein 96
hAAT	human α_1_-antitrypsin
HSP70	heat shock protein 70
HVA	high-voltage-activated (calcium channel)
IACUC	Institutional Animal Care and Use Committee
IL-1β	interleukin-1β
IL-6/8/10	interleukin-6/8/10
i.p. (IP)	intraperitoneal(ly)
i.pl.	intraplantar
LPS	lipopolysaccharide
LRP1	low-density lipoprotein receptor-related protein 1
LVA	low-voltage-activated (calcium channel)
Na_V_	voltage-gated sodium channel (isoforms Na_V_1.6–Na_V_1.9)
NF-κB	nuclear factor κB
NGF	nerve growth factor
NSAIDs	non-steroidal anti-inflammatory drugs
P2X4	P2X purinoceptor 4 (ATP-gated ion channel)
PKA	protein kinase A
RMP	resting membrane potential
SCN9A/SCN10A/SCN11A	genes encoding Na_V_1.7/Na_V_1.8/Na_V_1.9
SEM	standard error of the mean
SERPIN	serine protease inhibitor
SNI	spared nerve injury
SP16	α_1_-antitrypsin C-terminus–derived LRP1-agonist peptide
TLR2/4	Toll-like receptor 2/4
TNF-α	tumor necrosis factor-α
TRP	transient receptor potential (channel)
TTA-P2	selective T-type (Cav3.2) calcium channel blocker
TTX	Tetrodotoxin
VTD	Veratridine (pan Na_V_ channel modifier)
